# PTEN suppresses axon outgrowth by down-regulating the level of detyrosinated microtubules

**DOI:** 10.1371/journal.pone.0193257

**Published:** 2018-04-04

**Authors:** Christina Kath, Paloma Goni-Oliver, Rainer Müller, Carsten Schultz, Volker Haucke, Britta Eickholt, Jan Schmoranzer

**Affiliations:** 1 Charité –Universtiätsmedizin, Virchowweg 6, Berlin, Germany; 2 Leibniz Institute for Molecular Pharmacology, Robert-Roessle-Strasse 10, Berlin, Germany; 3 European Molecular Biology Laboratory, Meyerhofstraße 1, Heidelberg, Germany; University of Illinois at Chicago, UNITED STATES

## Abstract

Inhibition of the phospholipid phosphatase and tumor suppressor PTEN leads to excessive polarized cell growth during directed cell migration and neurite outgrowth. These processes require the precise regulation of both the actin and microtubule cytoskeleton. While PTEN is known to regulate actin dynamics through phospholipid modulation, whether and how PTEN regulates microtubule dynamics is unknown. Here, we show that depletion of PTEN leads to elevated levels of stable and post-translationally modified (detyrosinated) microtubules in fibroblasts and developing neurons. Further, PTEN depletion enhanced axon outgrowth, which was rescued by reducing the level of detyrosinated microtubules. These data demonstrate a novel role of PTEN in regulating the microtubule cytoskeleton. They further show a novel function of detyrosinated microtubules in axon outgrowth. Specifically, PTEN suppresses axon outgrowth by down-regulating the level of detyrosinated microtubules. Our results suggest that PTEN’s role in preventing excessive cell growth in cancerous and neurodevelopmental phenotypes is partially exerted by stabilization and detyrosination of the microtubule cytoskeleton.

## Introduction

Tissue development and repair require the precise and local regulation of cytoskeletal dynamics to establish cell polarity for directed cell growth or migration. A large number of regulatory factors, including G-protein coupled receptors, receptor tyrosine kinases, Rho-family GTPases and phospholipid modifiers, are known to orchestrate both the actin and the microtubule (MT) cytoskeleton during these processes [1–4]. The phospholipid phosphatase and tumor suppressor PTEN (phosphatase and tensin homologue deleted on chromosome ten) was previously identified as a negative regulator of cell motility [5–7]. While enhanced cell migration and proliferation are hallmarks of cancerous phenotypes in PTEN mutants, PTEN has also emerged as a key regulator of neuronal development, with roles in a large variety of overgrowth and developmental disorders, including cancer and neuropathies [8–11]. However, the precise cellular mechanisms of how PTEN regulates polarized cell growth are unclear.

To date, most studies that investigated the role of PTEN and the cytoskeleton in cellular growth focused on actin dynamics. For instance, during directed cell migration of *Dictyostelium*, neutrophils or fibroblasts, the reciprocal localization and activity of PTEN and its antagonist, the phosphoinositide 3-kinase (PI3K), induce local cell growth by regulating the cortical actin cytoskeleton [6,12–14]. The antagonistic activity of PTEN and PI3K locally catalyze the lipid products phosphatidylinositol 4,5-bisphosphate PI(4,5)P_2_ (short PIP2) and PI(3,4,5)P_3_ (short PIP3), of which the latter triggers actin-driven membrane protrusions at the growing cell edge. In developing neurons the balanced activities of PTEN and PI3K are essential for many aspects of neuronal polarity, including neuronal migration as well as neurite outgrowth and specification. Specifically, PTEN inhibition promotes neurite outgrowth in several systems in an actin dependent manner [15,16].

PTEN has an important role in down-regulating the PI3K-PIP3/Akt signaling pathway that involves several signaling kinases (i.e. Akt, GSK3β) and small GTPases (i.e. Rho-family) [9,14,17]. Specifically, the inhibition of PTEN leads to an inactivation of the key signaling kinase GSK3β by up-regulation of PI3K-PIP3/Akt signaling [2,18,19]. Inactivation of GSK3β is also known to enable the formation of stable and detyrosinated MTs in fibroblasts [20]. However, it remains unknown whether and how PTEN might regulate the dynamics of the MT cytoskeleton. Therefore, we hypothesized that PTEN might function in regulating the MT cytoskeleton and through that contribute to polarized cell growth, for example during neuronal development.

A hallmark of MTs is that the majority undergo dynamic instability, a cycle between filament growth, pause and shrinkage [21,22]. During directed cellular growth a subset of these MTs becomes stabilized (i.e. long-lived) toward the growing cell edge, including the leading edge of migrating cells or the growth cone of neurites [23,24]. This selectively stabilized MT pool is implicated in promoting local cell growth by enhancing directed traffic toward the growing cell edge [2]. While it is well established that the local balance between labile (i.e. dynamic) and stable MT filaments is crucial for the establishment and maintenance of directed membrane traffic and cellular growth [25–29], how this balance is controlled has remained unclear.

One cardinal feature of the stable MT pool is that it is enriched in posttranslational modifications via the action of several MT-targeted enzymes [27,30,31]. Detyrosination, the removal of the C-terminal tyrosine of alpha-tubulin, is a posttranslational modification that predominantly occurs on stable MTs. The enzyme causing detyrosination remains unknown, while the tubulin tyrosine ligase that re-ligates the tyrosine residue by acting on the soluble tubulin pool, has been identified [32,33]. By using a specific antibody against detyrosinated alpha-tubulin, both lamellipodia of migrating cells as well as the axons of developing neurons were found to be highly enriched in detyrosinated MTs, marking the stable pool of MTs [23,24,34,35]. Several studies support the idea that detyrosination enhances anterograde membrane traffic that could promote local cell growth [36–38]. However, the function of detyrosinated MTs, especially in local cell growth, remains elusive [27,31]. Moreover, it has not been investigated whether changes in PTEN activity affect the levels of stable and detyrosinated microtubules.

Here, we investigated the role of PTEN in regulating the dynamic and detyrosination state of the MT cytoskeleton, as well as its impact on axon outgrowth. We demonstrate that depletion of PTEN regulates MT stability and detyrosination through the PI3K-PIP3/Akt pathway. Specifically depletion of PTEN induces elevated levels of stable and detyrosinated MTs in both, migrating fibroblasts and developing hippocampal neurons. Interestingly, the enhanced axon outgrowth phenotype of PTEN-deficient neurons is rescued by reducing detyrosination, catalyzed by overexpression of the antagonistic tubulin tyrosine ligase. This demonstrates that detyrosination itself contributes to axon outgrowth, and that PTEN controls axon outgrowth by antagonistically regulating the level of detyrosinated MTs. Moreover, our results extend PTEN’s role to be a general cytoskeletal switch that, beside actin, also controls microtubules to prevent excessive cell growth.

## Results

### Depletion of PTEN enhances microtubule stability and detyrosination

Recent studies demonstrated that local cell growth critically depends on the precise regulation of the dynamics of both the actin and MT cytoskeletons [3,4]. Most studies that investigated the role of PTEN and the cytoskeleton in cellular growth focused on the effects of PTEN inhibition on promoting actin-based membrane remodeling. A functional link between PTEN and the dynamic state of MTs has not been investigated.

**Fig 1:**
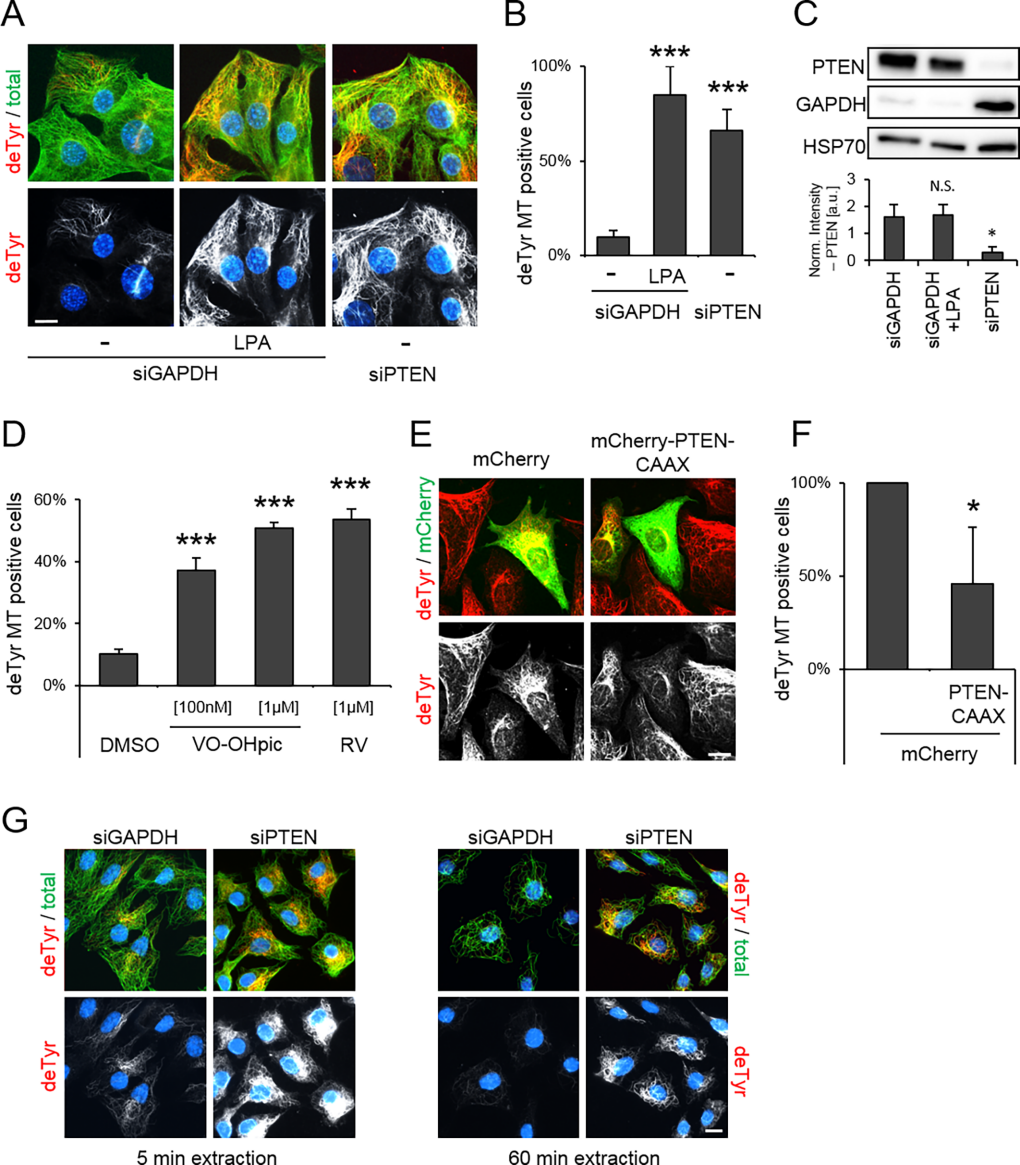
PTEN levels regulate detyrosination of microtubules. [A] NIH/3T3 cells were siRNA depleted of GAPDH (control) or PTEN, serum-starved and treated with 10 μM LPA (positive control) as indicated. Representative images of cells immunostained for detyrosinated tubulin (deTyr, red), total tubulin (green) and DNA (blue). [B] Percentage of deTyr positive cells depleted of GAPDH or PTEN. Error bar = StdDev, N = 5 (total of >150 cells each). [C] Cell lysates of siRNA-depleted cell were analyzed by western blotting against PTEN, GAPDH and HSP70 as indicated. Error bar = StdDev, N = 3. [D] Percentage of deTyr positive cells treated with PTEN inhibitors (VO-OHpic [100 nM, 1 μM], RV00I (RV) [1μM]) as indicated. Error bar = StdDev, N = 4 (total of >150 cells each). [E] NIH/3T3 cells were transfected with mCherry or mCherry-PTEN-CAAX, seeded on fibronectin-coated coverslips and immunostained against deTyr (red) and mCherry (green). [F] Percentage of deTyr positive cells, transfected with mCherry or mCherry-PTEN-CAAX, seeded on fibronectin-coated coverslips. Error bar = StdDev, N = 4 (total of > 50 cells each). [G] NIH/3T3 cells were siRNA depleted for GAPDH or PTEN and extracted (5 min or 60 min) prior fixation as indicated. Representative images of cells immunostained against detyrosinated tubulin (red), total tubulin (green) and DNA (blue). N = 3 (total of >100 cells each). Scale bars, 10 μm, * p<0.05, *** p<0.001.

First, we tested whether depletion of PTEN lead to changes in the level of stable MTs in NIH/3T3 fibroblasts, a well-established system to quantify the formation of stable MTs by immunofluorescence label for the detyrosination of the C-terminus of alpha-tubulin [34,35,39]. In this system serum-starved NIH/3T3 cells contain a low level (< 15%) of detyrosinated (i.e. stable) MTs since the known G-protein coupled receptor (GPCR) and Rho-GTPase pathway is inactive (Fig 1A and 1B). Addition of lysophosphatidic acid (LPA) activates the pathway leading to an increase in the number of cells (> 80%) that score positive for detyrosinated MTs. Here, we use LPA stimulation of serum-starved cells as a positive control for the formation of stable and detyrosinated MTs. The level of PTEN did not change upon LPA stimulation (Fig 1C). Interestingly, PTEN depletion via siRNA significantly increased the level of detyrosinated MTs in serum-starved cells to above 65% (Fig 1A and 1B and 1C; S1A and S1B Fig). To verify whether elevated levels of detyrosination are a reliable quantitative marker for MT stability and that PTEN depletion enhanced MT stability, we used two alternative assays for MT stability that rely on immunostaining for total or deTyr MTs in extracted or nocodazole treated cells, respectively [40]. Both extraction and nocodazole treatment prior fixation are known to deplete the dynamic, but not the stable, pool of MTs. Consistent with the results obtained in serum-starved cells, extracted and nocodazole treated cells depleted of PTEN both showed an elevation of stable MTs (Fig 1G, S1C Fig). Using an antibody against acetylation, another post translational modification of tubulin that has been associated with MT stability [27], did not show significant changes upon PTEN depletion (S1D and S1E Fig). Furthermore, treatment of the cells with known PTEN inhibitors (VO-OHpic, RV00I) increased the level of detyrosinated MTs up to 5-fold in a dose-dependent manner (Fig 1D). In analogy to the depletion experiments, elevated levels of PTEN should prevent the formation of detyrosinated MTs. To test this, we overexpressed PTEN in serum-grown NIH/3T3 cells that contain a high level of detyrosinated MTs. Both, the expression of membrane-targeted PTEN (mCherry-PTEN-CAAX, [41]) and cytosolic PTEN (GFP-PTEN) lead to a significant reduction of detyrosinated MTs in comparison to the control cells (Fig 1E and 1F; S8 Fig left). These results show that PTEN antagonistically regulates detyrosination and stability of MTs.

### PIP3 levels promote detyrosination of microtubules

**Fig 2:**
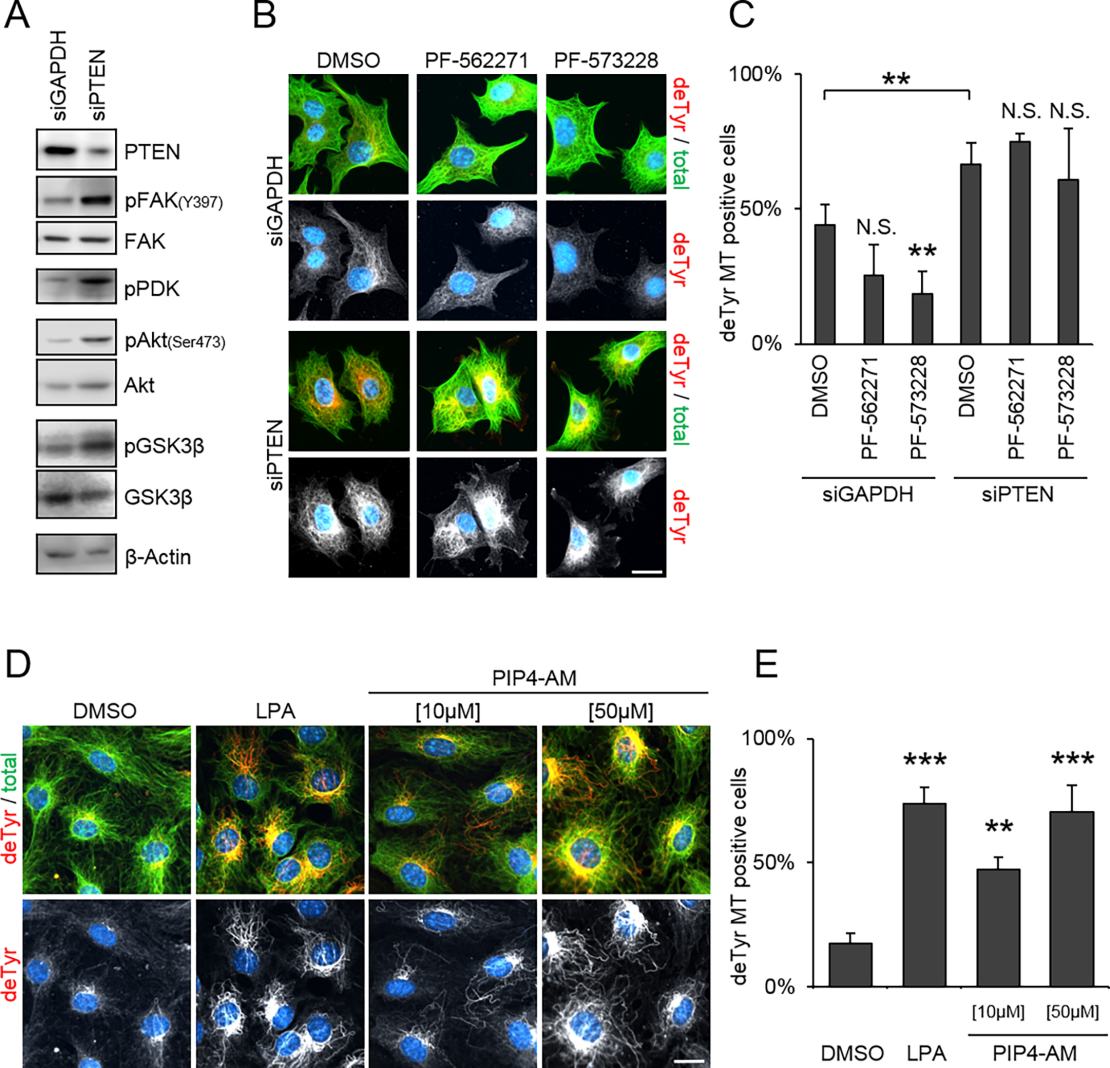
PI(3,4,5)P3 analog stimulates detyrosination of microtubules. **[A]** NIH/3T3 cells were siRNA depleted for GAPDH (control) or PTEN and serum depleted overnight. Cell lysates were analyzed by western blotting against PTEN, phospho-FAK (Y397), total FAK, phospho-PDK1, phospho-Akt (Ser473), total Akt, phospho-GSK3β, total GSK3β and β-Actin. Representative images of 3 independent experiments are shown. **[B, C]** NIH/3T3 cells were siRNA depleted against GAPDH or PTEN, treated with FAK inhibitors (PF-562271[1 μM], PF-573228 [10 μM]) as indicated and seeded on fibronectin-coated coverslips for 2 h. **[B]** Representative images of cells immunostained against, detyrosinated tubulin (red), total tubulin (green) and DNA (blue). **[C]** Percentage of detyrosinated microtubules positive cells. Error bar = StdDev, N = 4 (total of >150 cells each). **[D, E]** NIH/3T3 cells were serum depleted for 2 days and treated with LPA [10 μM] (positive control) or PIP4-AM as indicated. **[D]** Representative image of cells immunostained against detyrosinated tubulin (deTyr, red), total tubulin (green) and DNA (blue). **[E]** Percentage of deTyr positive cells. Error bar = StdDev, N = 3 (total of >150 cells each). Scale bars, 10 μm, ** p<0.01, *** p<0.001.

PTEN depletion is known to activate the PI3K/PIP3-Akt-GSK3β pathway [9,14,17]. We verified this in our cell system by using western blot analysis of lysates from PTEN depleted NIH/3T3 cells against known pathway components, observing an increase in activated (phosphorylated) PDK1 and Akt, as well as inactivated (phosphorylated) GSK3β (Fig 2A, S3 Fig). Since inactivation of GSK3β is known to induce stable and detyrosinated MTs [20] these data strongly suggest that depletion of PTEN stabilizes MTs by activation of the PI3K-Akt-GSK3β signaling pathway. By using a chemical inhibitor of GSK3β (SB216763) in cells overexpressing GFP-PTEN we verified that GSK3β acts downstream of PTEN (S5 Fig). We next investigated the upstream factors that could contribute to the activation of this pathway by PTEN depletion. The phosphatase PTEN is known for its ability to dephosphorylate both lipids and proteins. Specifically, PTEN preferentially dephosphorylates PIP3 [42–44]) but can also dephosphorylate, and thus inactivate, the focal adhesion kinase (FAK) [45]. FAK also has a functional relation to MTs, as it was previously shown that the formation of detyrosinated (stable) MTs in fibroblasts depends on FAK activation (i.e. phosphorylation) [39]. Conceivably, PTEN could regulate MT stability either by a PIP-dependent or a FAK-dependent switch. To test whether FAK signaling is essential for PTEN depletion-dependent detyrosination of MTs we performed a reseeding assay of serum-grown NIH/3T3 cells treated with FAK inhibitors (PF-562271, PF-573228) after PTEN depletion (Fig 2B and 2C). The effectiveness of the inhibitors was validated by western blotting for phosphorylated FAK (Y397) (S4 Fig). As expected, the control cells showed a significant reduction of detyrosinated MTs upon FAK inhibition. If FAK were essential for MT detyrosination in PTEN depleted cells, then FAK inhibition would prevent the elevated level of detyrosinated MTs. However, PTEN depleted cells showed no significant changes in their elevated level of detyrosinated MTs upon FAK inhibition. This result demonstrated that FAK signaling does not seem to be essential for MT stabilization mediated by PTEN depletion. Alternatively, depletion or inhibition of PTEN could mediate MT stabilization by elevating the level of PIP3. If so, acutely increasing the level of PIP3 in serum-starved cells would phenocopy PTEN depletion. Indeed, adding the functional PIP3 analog PIP4-AM [46] to serum-starved cells increased the percentage of detyrosinated MT positive cells in a dose-dependent manner up to 70%, similar to the maximum level of detyrosinated MTs reached upon control (LPA) stimulation (Fig 2D and 2E). These results show that PIP3 signaling, rather than FAK signaling, is the dominant switch that leads to an elevated level of stable MTs in cells depleted of PTEN.

### PTEN regulates detyrosination of microtubules and axon length in neurons

**Fig 3:**
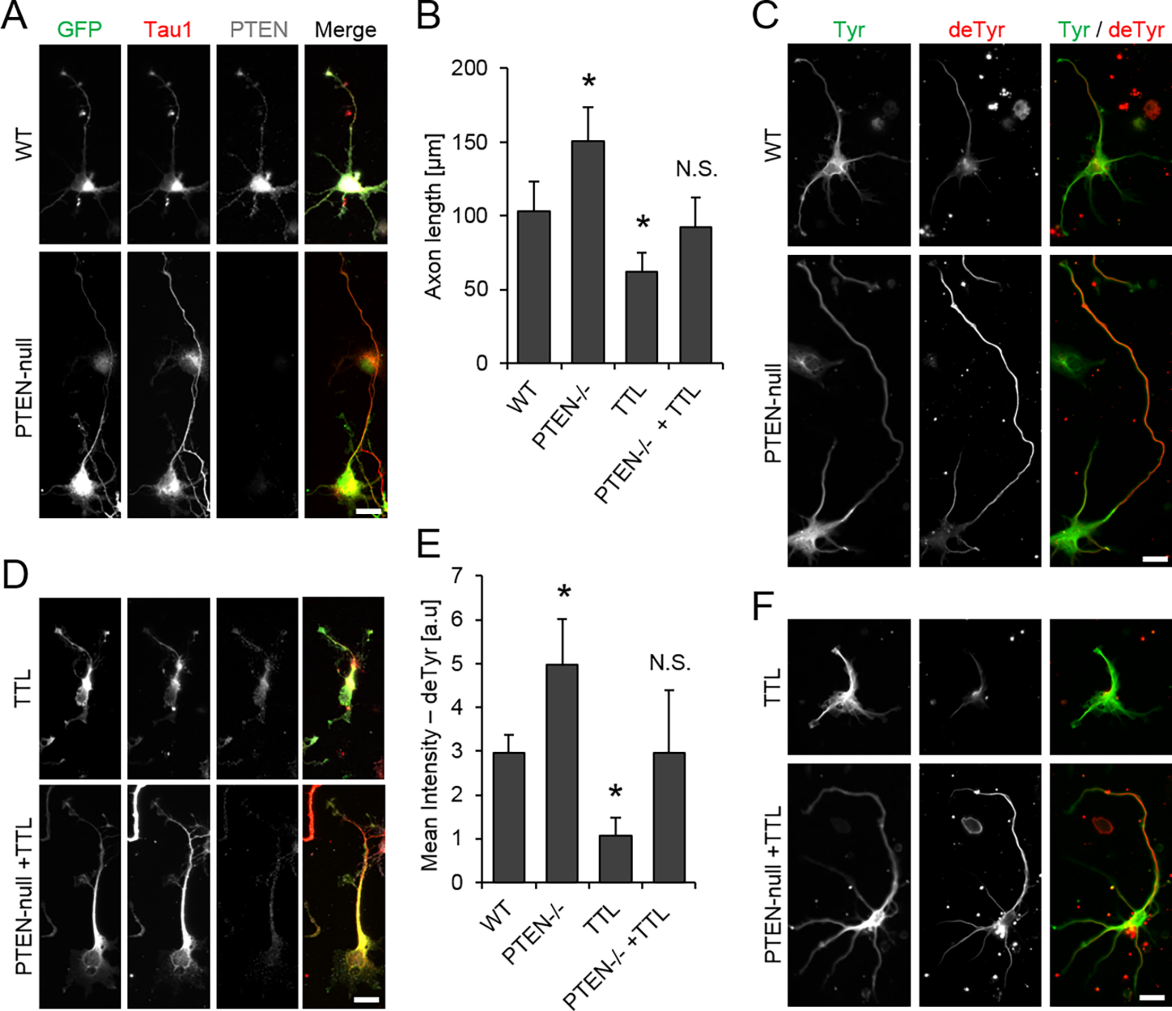
Detyrosination of microtubules and axon length is enhanced by loss of PTEN in neurons. Hippocampal neurons isolated from *PTEN*^*flox/flox*^ E16 mice were transfected with RFP-IRES-Cre (PTEN-null) and/or GFP-tubulin tyrosine ligase (TTL) at DIV 0, seeded on PLL coated coverslips and fixed at DIV 3. **[A, D]** Representative images of neurons immunostained against GFP (green), Tau1 (red) and PTEN (grey). Scale bar, 20 μm. **[B]** Quantification of axon length. Error bar = StdDev, N = 4 (total of >100 cells each). **[C, F]** Representative images of neurons immunostained against detyrosinated- (red) and tyrosinated- (green) tubulin. **[E]** Mean intensity of the detyrosination signal in RFP (PTEN-null) or GFP positive (GFP-TTL) neurons. Error bar = StdDev, N = 5 (total of >100 cells each). Scale bars, 20 μm, * p<0.05.

Most previous studies that investigated the role of PTEN in regulating the cytoskeleton during neuronal development focused on effects on the actin cytoskeleton. In several model systems PTEN inhibition promotes actin-based neurite outgrowth [15,16]. Whether PTEN deficiency could affect the dynamic state of the MT cytoskeleton and that way contribute to neurite outgrowth has not been investigated. To address this question, we quantitatively assayed MT stabilization and detyrosination in PTEN depleted developing neurons. We used isolated mouse embryonic hippocampal neurons from PTEN^flox/flox^ E16 mice transfected with RFP-IRES-Cre (PTEN-null) [47]. The high efficiency of PTEN depletion in RFP positive cells was verified by immunostaining against endogenous PTEN (S6 Fig). As expected, PTEN-null neurons (DIV 3) displayed elongated (50% longer) axons marked by immunostaining against Tau protein (Fig 3A and 3B). Consistently, strongly elevated levels of PTEN by overexpression of GFP-PTEN in wild-type neurons prevented the outgrowth of axons (S5 Fig). This PTEN-mediated block of axon outgrowth was ineffective in neurons treated with a GSK3β inhibitor (SB216763) showing that, as mentioned earlier, PTEN acts upstream of GSK3β (S5 Fig). Importantly, PTEN deficient neurons displayed an enhanced level of detyrosinated MTs (Fig 3C and 3E). Vice versa, the accumulation of detyrosinated MTs was prevented in PTEN overexpressing neurons (S5 Fig). These data demonstrate that PTEN negatively regulates the level of detyrosinated MTs and that detyrosination correlates positively with elongated axons. This suggests a causal relationship between detyrosination and axon outgrowth.

### PTEN suppresses axon outgrowth by down-regulating detyrosination of microtubules

The detyrosination of the C-terminus of alpha tubulin can be reverted by overexpression of the tubulin tyrosine ligase (TTL) [48]. To investigate whether detyrosination itself affects axon outgrowth, we used TTL as an enzymatic tool to reduce the level of detyrosinated MTs in developing neurons. In a previous study, which focused on the structural aspects of TTL, it was shown that changing the level of TTL in developing neurons negatively correlated with the length of the neurites [49]. Whether the level of TTL correlates with that of detyrosinated MTs was not shown. Consistent with a negative correlation between TTL and detyrosination levels, we observed that depletion of TTL in NIH/3T3 cells elevated the level of detyrosinated MTs (S7 Fig). Further, overexpression of GFP-TTL strongly reduced the level of detyrosinated MTs and prevented axon outgrowth (Fig 3B, 3D, 3E and 3F). TTL acts directly on tubulin while PTEN acts at the membrane. Therefore, it would be expected that PTEN acts upstream of TTL in the formation of detyrosinated MTs. Consistent with this, overexpression of PTEN in TTL depleted fibroblasts did not alter the level of detyrosination, demonstrating that TTL indeed acts downstream of PTEN (S8 Fig). To further test whether detyrosination was necessary for the elongated axon phenotype, we assayed both the level of detyrosinated MTs and the extent of axon outgrowth upon GFP-TTL expression in PTEN deficient neurons (Fig 3B, 3D, 3E and 3F). Interestingly, both phenotypes observed in PTEN deficient neurons, the elevated level of detyrosination and the elongated axons, were rescued upon expressing GFP-TTL (Fig 3F). These data demonstrate that the detyrosination of MTs itself is necessary for enhancing axon outgrowth. Moreover, they show that PTEN suppresses axon outgrowth by down-regulating the level of detyrosinated MTs.

## Discussion

**Fig 4:**
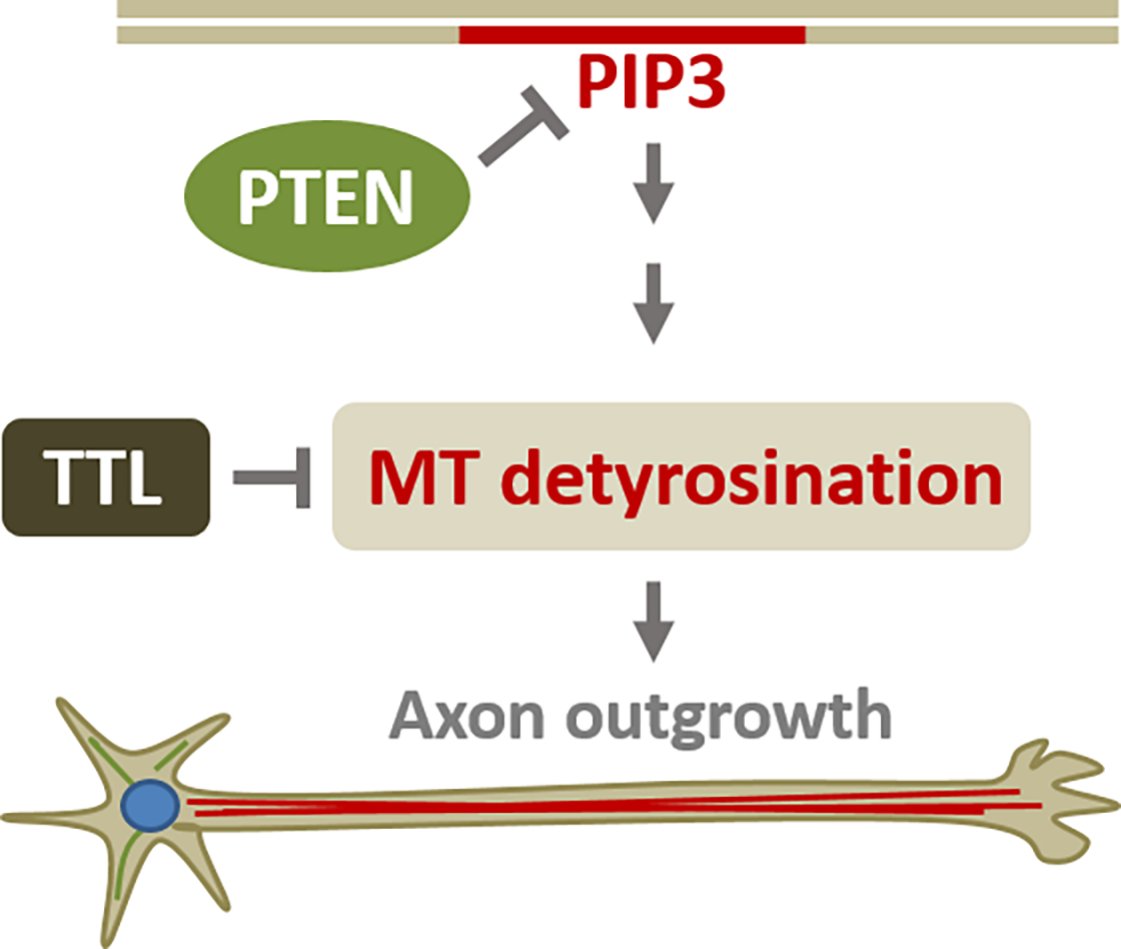
Model of PTEN regulation of axonal outgrowth through detyrosination of microtubules. During steady state conditions, PTEN downregulates the PIP3 (Akt/GSK3β) signaling pathway and that way prevents excessive cell growth. Here, we show that depletion of PTEN leads to an elevated level of stable and detyrosinated microtubules via upregulation of the PIP3 (Akt/GSK3β) pathway. This causes excessive cell growth (i.e. axonal outgrowth) that is prevented by reverting the detyrosination of MTs through overexpression of the tubulin tyrosine ligase (TTL). The plasma membrane is indicated with a grey double line (top) with the PIP3 domain in red. Detyrosinated MTs within the axon of a developing neuron are indicated in red.

Tissue development and repair require the precise and local regulation of cytoskeletal dynamics to establish cell polarity and direct cell growth. A large number of regulatory factors that orchestrate both the actin and the microtubule (MT) cytoskeleton during processes that require localized cell growth, such as directed cell migration and neurite outgrowth, are required [1,2]. PTEN has emerged as one of the key players in regulating directed cell growth, however the extent of the cellular mechanisms by which PTEN regulates the cytoskeleton is unclear. In directionally migrating cells PTEN is known to down-regulate the PI3K/PIP3 signaling pathway that triggers local cell growth through the actin cytoskeleton. In neuronal cells, little is known about the role of PTEN in regulating the cytoskeleton. Notably, the potential roles of PTEN in regulating the MT cytoskeleton have not been investigated. Here, we demonstrate that PTEN negatively regulates both stabilization and detyrosination of MTs in fibroblasts and primary neurons. We further demonstrated that elevated detyrosination of MTs induced by lack of PTEN enhances axon outgrowth. These data establish a novel role of PTEN as a cytoskeletal switch that, in addition to known actin-based processes, regulates the dynamics of the MT cytoskeleton to contribute to local cell growth and morphology (Fig 4).

While it is clear that detyrosination accumulates on stable MTs both in migrating fibroblast and growing axons, the precise function of detyrosinated MTs, especially in local cell growth, remains elusive [27,31]. Recent studies support the view that detyrosination of MTs enhances anterograde membrane traffic which could be supportive for local cell growth [36–38]. However, the role of detyrosination in neuronal development remained unclear. Here we showed, using a detyrosination specific antibody, that detyrosination positively correlates with the extent of axon outgrowth. Moreover, the elongated axon phenotype upon loss of PTEN was reverted by reducing the level of detyrosination. These results establish a novel role of detyrosination of MTs in directed cell growth, specifically in axon outgrowth (Fig 4). Our results are consistent with the view that detyrosination enhances anterograde membrane traffic towards the growing end of the cell, and this way promotes local cell growth. Whether the detyrosination-regulated cell growth depends on enhanced kinesin-based anterograde traffic [36–38] or enhanced MT stability [50] will be an interesting question for future studies.

The precise mechanism on how dynamic MTs are captured and stabilized near the membrane is still unclear [1]. Current models include a mechanism in which MT stabilization (detyrosination) in fibroblasts requires the activation of FAK as well as a G-protein coupled receptor signaling pathway [35,39]. Another model favors a PIP-switch that leads to transient to long-term stabilization by phosphoinositides (PIPs) and MT-binding proteins [51]. Consistent with the FAK-dependent model, both FAK activation and the level of detyrosinated MTs increased upon depletion of PTEN. However, when we chemically inhibited FAK activation (Y397), we did not see a reduction of stable and detyrosinated MTs. Thus, FAK alone cannot be responsible for detyrosination in PTEN depleted cells. Instead, treating serum-starved cells that normally have a low level of detyrosinated MTs with a PIP3-analog induced detyrosinated MTs. These data strongly suggest that PIP3 signaling, rather than FAK signaling, is the dominant pathway component that leads to an elevated level of stable (detyrosinated) MTs in cells depleted of PTEN (Fig 4). Our results are consistent with the view that long-lived MTs are generated through PIP3-binding proteins [51].

It has previously been shown that GSK3β inhibition leads to an elevated level of detyrosinated MTs in migrating fibroblasts [20]. Here we show that both PTEN depletion and GSK3β inhibition increase detyrosination levels and that this elevated level of detyrosination promotes outgrowth of developing axons (Fig 3, S5 Fig). These results are in agreement with the finding that neurons from mice lacking TTL showed enlarged growth cones and enhanced branching [52]. Most recently, the growth promoting function of detyrosination in neurons was confirmed in a study that identified the enzymatic factors responsible for tubulin detyrosination [53]. Specifically, downregulation of vasohibins and an associated factor depleted detyrosination in cultured neurons and delayed axon outgrowth and differentiation [53]. Future investigations might explain the controversy between the above data and studies that observed that downregulating detyrosination promoted nerve regeneration [54–56].

In addition to its role as a tumor suppressor, PTEN plays crucial roles in the central nervous system, both during brain development and in adulthood. De-regulation of PTEN has been attributed to defects in neurogenesis, neurite outgrowth, synaptogenesis, and synaptic plasticity [9,11,17]. Given the important role of PTEN in neuronal development, it is not surprising that several PTEN mutations are associated with clinical syndromes (i.e. Cowden, Bannayan-Riley-Ruvalcaba, Proteus) and neurological disorders (i.e. autism spectrum disorders) that are based on neurodevelopmental defects [10,11]. Many of these PTEN-associated disorders are associated with cellular overgrowth defects, including enlarged cell size and macroencephaly [55–58]. However, the cellular mechanisms of how PTEN inhibition causes excessive cellular growth, especially regarding the cytoskeleton, are still unclear. Several studies suggest links between the deregulation of PTEN and actin-based processes that could contribute to cell growth. We found that PTEN-deregulation leads to distinct changes in the MT cytoskeleton (i.e. stabilization, detyrosination) that are causative for excessive directed cell growth (Fig 4). This novel link between PTEN and the MT cytoskeleton extends PTEN’s role to be a general cytoskeletal switch that, beside actin, also controls microtubules to prevent excessive directed cell growth. It will be interesting to investigate the relative importance of PTEN’s regulation of the actin versus the MT cytoskeleton, including potential modes of cytoskeletal crosstalk, to advance the understanding of PTEN-related over-growth phenotypes and associated neurodevelopmental disorders.

## Materials and methods

### Cell culture and siRNA depletion

NIH/3T3 cells were cultured in low glucose Dulbecco’s modified Eagle’s medium (DMEM) supplemented with 10% (v/v) heat-inactivated bovine calf serum (BCS), and routinely kept at 37°C and 5% CO2. For transient depletion (knockdown) of endogenous protein in NIH/3T3, two rounds of siRNA forward transfection were performed in 6-well plates using RNAiMAX (Life Technologies). The following siRNAs were used: GAPDH (5’-AAA GUU GUC AUG GAU GACC-3‘), pool of PTEN#1 (5’-GAU GGA UUC GAC UUA GAC UUG-3‘) and PTEN#2 (5’-CAA UAG GAC AUU GUG UCA GAU-3‘), TTL (5‘-CAU UCA GAA AGA GUA CUCA-3‘). Cells were plated in a 6-well plate to be 80% confluent on day 1 of transfection. For one 6-well, 2 μl of siRNA [100μM] were added into 250 μl Opti-MEM and gently mixed. In parallel, 6.25 μl of RNAiMAX were added into 250 μl Opti-MEM and gently mixed. Both, the diluted siRNA and transfection medium were mixed together in the 6-well plate and incubated for 5–10 min at room temperature. Meanwhile, cells were washed 2 times with PBS and detached from the culture dish by using Trypsin-EDTA (1 ml for a 10 cm dish, Gibco, Invitrogen, Carlsbad, U.S.A.) for 5 min at 37°C. The cells were re-suspended in 10 ml of culture medium containing BCS to inactivate the trypsin. Cell density was determined by counting in a Neubauer chamber. 0.5 x 10^5^ cells were diluted in 1.5 ml culture medium containing BCS and added on top of the diluted siRNA–RNAiMAX mixture to a final volume of 2 ml. On day 2 the transfection medium was exchanged by culture medium. On day 3 we repeated the procedure for a second round of transfection. For western blot experiments, one 6-well dish was split into two 6-well dishes. For immunofluorescence assays, one 6-well dish was split into four 12-well dishes containing glass coverslips. On day 4, depending on the experiment, the medium was changed and the cells were starved in DMEM without BCS, respectively.

### Protein expression

Transient (over)expression of fluorescently labeled proteins in NIH/3T3 was performed by electroporation using the Nucleofector 2b device (AMAXA). The following inserts/plasmids were used: GFP/VECTOR, GFP-PTEN/VECTOR, mCherry-PTEN-CAAX/Vector [41] and GFP-TTL (kind gift from C. Hoogenraad laboratory, University Utrecht, Netherlands). Cells were washed 2 times with PBS and detached from the culture dish by using Trypsin-EDTA (1 ml for a 10 cm dish, Gibco) for 5 min at 37°C. The cells were re-suspended in 10 ml of culture medium containing BCS to inactivate the trypsin. Cell density was determinate by counting in a Neubauer chamber. Cells [1 x 10^6^] were centrifuged for 5 min at 5.000 RPM. The cell pellet was re-suspended in 100 μl electroporation buffer and 5 μg of DNA added. The cell—DNA mixture was transferred into the electroporation cuvette and electroporation was performed by using the A-024 program. The treated cells were diluted in 2 ml pre-warmed culture medium containing BCS an added into two 6-well plate dishes containing fibronectin coated glass coverslips. After one hour, the cells were nearly fully attached to the fibronectin coated coverslips. To remove dead cells, the culture medium was changed.

### Serum starvation

For serum starvation, NIH/3T3 cells were washed 3 times in serum-free DMEM and kept in 1 ml total volume in 3.5 cm (or 6-well) dishes of serum-free DMEM for 2 days.

### PTEN inhibitor study

PTEN inhibitors Vo-OHpic [100 nM, 1 μM], RV00I (RV) [1 μM] dissolved in dry DMSO. Cells were starved for 2 days in serum-free DMEM and treated with DMSO (control) or PTEN inhibitors as indicated for 2 h.

### FAK inhibitor study

FAK inhibitors (PF-562271, PF-573228) obtained from ApexBio were dissolved in dry DMSO. Cells were treated with DMSO (control) or FAK inhibitors as indicated for 1 h and re-seeded on fibronectin coated coverslips for 2 h.

### GSK3β inhibitor study

Hippocampal neurons were transfected with GFP or GFP-PTEN at DIV 0 using electroporation (AMAXA, Nucleofector 2b device). Seeded on poly-(L-lysine)-coated glass coverslips. For inhibitor treatments, GSK3β inhibitor SB216763 [50 nM, 100 nM] were dissolved in dry DMSO and neurons treated from DIV 1 to DIV 3.

### PIP4/AM experiments

Cell-permeable acetoxymethyl ester (AM)-protected phosphatidyl-inositol derivatives were synthesized at the laboratory of C. Schultz (EMBL, Heidelberg, Germany) as previously described [46]. For treatment of cells, PIP4/AM was dissolved in dry DMSO. A small portion of this PIP4/AM stock was diluted in serum-free medium to a final concentration of 10 and 50 μM. For immunocytochemistry, cells were seeded on coverslips, treated with DMSO (control) or PIP4/AM for 2 h at 37°C and then processed as described above.

### MT stability assays

In addition to the immunostain against deTyr tubulin we used two alternative MT stability assays, based on extraction and nocodazole treatment prior fixation [40]. For extraction cells were incubated with extraction buffer (PEM buffer (80 mM PIPES pH 6.9, 2 mM MgCl2 and 0.5 mM EGTA) + 0.2% TritonX-100) for 5 or 60 min at 37°C and fixed with MeOH (-20°C, 5 min). For nocodazole treatment cells were incubated with nocodazole (Sigma) (0.66 μM) for 30 min at 37°C and fixed with MeOH (-20°C, 5 min).

### Preparation of acid washed coverslips

Acid washing is used to enhance the cleanliness of the coverslip and etches make the glass better attachable for cells. Coverslips were placed in ceramic holders and incubated in 1M HCl for at least one hour. After 3 wash steps in ddH_2_O, coverslips were incubated 100% acetone for at least one hour. Followed by 3 wash steps in ddH_2_O. Finally, the coverslips were incubated in 70% and 100% EtOH for at least one hour, respectively. After drying, the coverslips were ready to use.

### Preparation of poly-L-lysine (PLL) coated coverslips

For a better cell attachment, acid washed, sterile coverslips were coated with sterile 100 μg/ml PLL, which provides a positive charge on the surface. Coverslips were placed in 12-well plates and covered with 100 μl of PLL solution and incubate for 1–2 h at room temperature. After incubation, the solution were aspirated and washed 2 times with water. The coverslips were dried in a sterile environment before use.

### Fibronectin coating

To enhance the attachment of NIH/3T3 cells or to trigger out-side-in integrin signaling, acid washed coverslips were coated with the extracellular matrix protein fibronectin. Fibronectin (Roche, Basel, Switzerland) in a final concentration of 1 mg/ml in ddH_2_O was incubated on coverslips for one to two hour at room temperature using the “sandwich” method. 22 x 22 mm Coverslips were placed on the ground of a 6-well dish and 80 μl fibronectin solution pipetted on each. The sandwich was prepared by covering each coverslip by a second coverslip. After incubation time, the sandwich was opened by flooding the coverslips with PBS and washed once more with PBS before use.

### Neuron cell culture

Hippocampal neurons isolated from *PTEN*^*flox/flox*^ E16 mice were transfected with RFP-IRES-Cre (PTEN-null) and/or GFP-TTL (TTL) at DIV 0 using electroporation (AMAXA, Nucleofector 2b device). Seeded on poly-(L-lysine)-coated glass coverslips and fixed at DIV 3. For inhibitor treatments, inhibitors were dissolved in DMSO and neurons treated from DIV 1 to DIV 3.

### Immunocytochemistry

NIH/3T3 cells grown on glass coverslips were washed with PBS and fixed in methanol (5 min, -20°C). Neurons grown on poly-(L-lysine)-coated glass coverslips were washed with PBS and fixed in 4% paraformaldehyde (PFA) for 10 min at room temperature. PFA fixed cells were permeabilized in PBS + 1% BSA supplemented with 0.3% Triton-X-100. Coverslips were blocked with PBS + 1% BSA for 30 min at room temperature. Antibodies were diluted into PBS + 1% BSA and the staining was performed in a tinted humid chamber. Primary antibody solution was incubated either for 1–2 h at room temperature or overnight at 4°C, then washed 3 times in PBS. Secondary antibody labeling followed the same procedure. After washing the coverslips were mounted with Immu-Mount (Waltham, Massachusetts, U.S.A.) on microscope slides.

### Antibodies

The following primary antibodies were used in this study: deTyr-Tubulin (rabbit, G.G.Gundersen, Columbia University, New York, USA), total Tubulin (mouse, clone B5-1-2, Sigma), Tubulin tyrosine ligase (rabbit, ProteinTech), PTEN (rabbit, clone 138G6, Cell Signaling), Tau1 (mouse, clone PC1C6, Chemicon), phospho-FAK (Y397) (rabbit, clone D20B1, Cell Signaling), total FAK (rabbit, clone D2R2E, cell signaling), phospho-PDK1, (rabbit, clone C49H2, Cell Signaling), phospho-Akt (Ser473), (rabbit, clone D9E, Cell Signaling), total Akt (rabbit, clone C67E7, cell signaling), phospho-GSK3β, (rabbit, cell signaling), total GSK3β (mouse, clone 7/GSK-3b, BD transduction), β-Actin (mouse, clone AC-15, Sigma), GAPDH (mouse, clone 71,1, Sigma), GFP (chicken, abcam), acytelated Tubulin (mouse, clone 6-11B-1, Sigma), tyrosinated Tubulin (rat, clone YL1/2, abcam) and GM130 (rabbit, clone EP892Y, abcam). Secondary antibodies for immunofluorescence labeled with Alexa Fluor dyes (488, 568 and 647) were purchased from Invitrogen. HRP-coupled antibodies for immunoblotting were from Dianova/Jackson ImmunoResearch.

### Fluorescence microscopy

Samples were imaged on Nikon Ti Eclipse—epifluorescence microscope (Nikon, Düsseldorf, Germany) equipped with a mercury arc lamp, a 40x oil immersion objective (NA 1.3) and standard filter sets (AHF Analysentechnik) to separate the Alexa Fluor dyes.

### Immunoblotting

Cells were lysed on ice for 30–60 min in lysis buffer (2% Triton X-100, 20 mM HEPES, pH 7.4, 100 mM KCl, 4 mM MgCl_2_, 1 mM PMSF, 0.03% protease inhibitor mixture (Sigma)). Cell lysates were analyzed by SDS-PAGE and immunoblotting. Western blots where imaged using the Odyssey FC system (LI-COR Biosciences, Lincoln, NE, U.S.A.) and the band densities were quantified using ImageJ.

### Quantification of deTyr positive cells

The percentage of detyrosinated MT positive cells was determined as previously described [23,59]. In brief, the fixed and immunostained cell monolayers were viewed by eye on a wide-field fluorescence microscope (Nikon, Ti Eclipse, 40x, 1.3 N.A.) on a cell-by-cell basis and scored for the presence of a significant number of microtubules (>10) that were brightly and clearly labeled with the antibody against deTyr tubulin. This way, a statistically large number of cells (> 100) could be efficiently scored for each condition. This method yielded very similar results to measuring the total intensity of the detyrosination fluorescence signal on the cell-by-cell basis (S2 Fig).

### Quantification of axon length and level of detyrosination

Neurons were co-immunostained for Tau1 as axon marker, imaged using the fluorescence microscope (Nikon, Ti Eclipse, 40x, 1.3 N.A.). The length of the axon was measured using the “segmented line” tool in ImageJ. For quantification of the detyrosination levels in axons, neurons were immunostained for deTyr tubulin and for the GFP-tag of the expressed GFP-fusion protein, imaged using the same fluorescence microscope (Fig 3D and 3F; S6 Fig). The detyrosination signal was quantified in ImageJ by measuring the signal inside a regional mask after thresholding on the basis of the GFP signal.

### Data analysis and statistics

Error bars represent the standard deviation (StdDev, SD) of at least three independent experiments. The statistical significance was assessed by using ANOVA with Bonferroni's Multiple Comparison Test (GraphPad Prism).

## Supporting information

S1 Fig Loss of PTEN induces elevated levels of stable and detyrosinated microtubules. **[A]** NIH/3T3 cells were siRNA treated with siGAPDH (control), siPTEN#1, siPTEN#2 or pooled siPTEN#1 + #2. Representative images of cells immunostained against, detyrosinated tubulin (red), total tubulin (green) and DNA (blue). **[B]** Cell lysates of siRNA-depleted cell were analyzed by western blotting against PTEN and HSP70 as indicated. Error bar = StdDev, N = 3 (* p<0.05, ** p<0.01). **[C]** NIH/3T3 cells were siRNA depleted for GAPDH, tubulin tyrosine ligase (TTL) or PTEN and treated with nocodazole (0.66 μM, 30 min). Representative images of cells immunostained against detyrosinated tubulin (red), total tubulin (green) and DNA (blue). **[D]** NIH/3T3 cells were siRNA depleted for GAPDH or PTEN. Representative image of cells immunostained against acetylated tubulin (green), detyrosinated tubulin (red) and DNA (blue). **[E]** Mean intensity of acetyl or deTyr positive cells. Error bar = StdDev, N = 3 (total of > 150 cells each, * p<0.05, ** p<0.01, *** p<0.001). Scale bars, 10 μm.
(TIF)

S2 Fig Comparison of different quantification schemes for deTyr positive cells. NIH/3T3 cells were siRNA depleted of GAPDH (control) or PTEN, serum-starved and treated with 10 μM LPA (positive control) as indicated. Cells were immunostained for detyrosinated tubulin (deTyr) and the fluorescence signal of each cell was measured. **[A]** Normalized average intensity of deTyr channel per cell without threshold. **[B]** Normalized average intensity of deTyr channel per cell with constant threshold. **[C]** Percentage of cells above the intensity threshold. **[D]** Percentage of deTyr positive cells by eye-scoring as in Fig 1B. N = 5 (total of >60 cells each). * p<0.05, ** p<0.01, *** p<0.001.
(TIF)

S3 Fig Changes in protein levels upon PTEN depletion. NIH/3T3 cells were siRNA depleted for GAPDH (control) or PTEN and serum depleted overnight. Cell lysates were analyzed by western blotting against PTEN, phospho-FAK (Y397), total FAK, phospho-PDK1, phospho-Akt (Ser473), total Akt, phospho-GSK3β, total GSK3β and β-Actin. Quantification of 3 independent experiments. Error bar = StdDev, N = 3 (* p<0.05, ** p<0.01).
(TIF)

S4 Fig Focal adhesion kinase (FAK) inhibitors do not change PTEN-mediated increase in detyrosination of microtubules. **[A, B]** NIH/3T3 cells were siRNA depleted against GAPDH or PTEN and treated with FAK inhibitors (PF-562271, PF-573228) as indicated and then seeded on fibronectin-coated coverslips for 2 h. **[A]** Cell lysates were analyzed by western blotting against pFAK and FAK. **[B]** Quantification of the western blot signal (ratio pFAK/FAK). Error bar = StdDev, N = 3 (** p<0.01, *** p<0.001).
(TIF)

S5 Fig Detyrosination of microtubules and axon length is reduced by overexpression of PTEN in neurons. Hippocampal neurons isolated from E16 mice were transfected with GFP or GFP-PTEN at DIV 0, seeded on PLL coated coverslips and fixed at DIV 3. **[A]** Representative images of neurons immunostained against GFP (green), PTEN (grey) and detyrosinated tubulin (deTyr, red). Scale bar, 50 μm. **[B]** Quantification of axon length in cells transfected with GFP or GFP-PTEN cells treated with GSK3β inhibitor (SB216763 [50 nM, 100 nM]) as indicated. Error bar = StdDev, N = 4 (total of >40 cells each, * p<0.05, *** p<0.001).
(TIF)

S6 Fig PTEN knockdown efficiency in neurons. Hippocampal neurons isolated from *PTEN*^*flox/flox*^ E16 mice were transfected with RFP-IRES-Cre (PTEN-null), GFP or GFP-tubulin tyrosine ligase (TTL) at DIV 0, seeded on PLL coated coverslips and fixed at DIV 3. After fixation, cells were immunostained against GFP and endogenous PTEN. The knockdown efficiency was assayed by scoring PTEN negative cells (low fluorescence intensity in the PTEN channel) in GFP positive cells. Error bar = StdDev, N = 3 (total of >30 cells each). This analysis was used in quantifying detyrosination in Fig 3.
(TIF)

S7 Fig TTL levels regulate detyrosination of microtubules. **[A]** NIH/3T3 cells were siRNA depleted of GAPDH (control) or tubulin tyrosine ligase (TTL) and serum depleted for 1 day. Representative image of cells immunostained against detyrosinated tubulin (red), total tubulin (green) and DNA (blue). Scale bar, 10 μm. **[B]** Cells were siRNA depleted as indicated. Percentage of detyrosinated microtubules positive cells. **[C]** Representative western blot of siRNA depleted cells as indicated. Error bar = StdDev, N = 4 (total of >150 cells each, *** p<0.001).
(TIF)

S8 Fig TTL regulates detyrosination of microtubules downstream of PTEN. **[A]** NIH/3T3 cells were siRNA depleted of GAPDH (control) or tubulin tyrosine ligase (TTL) and transfected with GFP, GFP-PTEN or mCherry-PTEN-CAAX and seeded on fibronectin-coated coverslips. Representative images of cells immunostained against detyrosinated tubulin (white), GFP/mCherry (magenta) and DNA (blue). Scale bar, 10 μm. **[B]** Cells were siRNA depleted as indicated. Percentage of detyrosinated microtubules positive cells. Error bar = StdDev, N = 3 (total of >100 cells each, *** p<0.001).
(TIF)
