# Supplementary figures and images for: PTEN suppresses axon outgrowth by down-regulating the level of detyrosinated microtubules
Source: PLoS One. 2018 Apr 4;13(4):e0193257. doi: 10.1371/journal.pone.0193257 (PMC5884485; doi:10.1371/journal.pone.0193257)

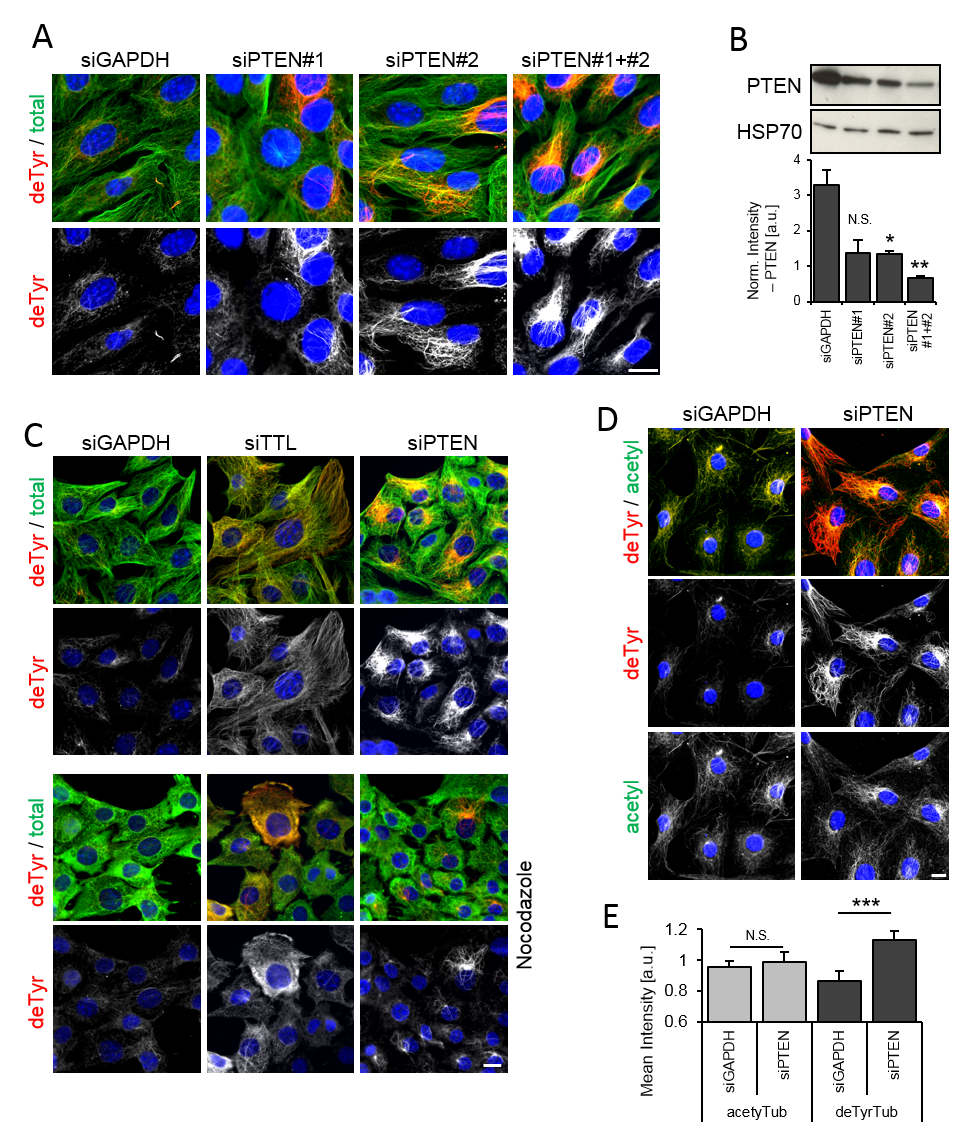

Supplement: S1 Fig — [A] NIH/3T3 cells were siRNA treated with siGAPDH (control), siPTEN#1, siPTEN#2 or pooled siPTEN#1 + #2. Representative images of cells immunostained against, detyrosinated tubulin (red), total tubulin (green) and DNA (blue). [B] Cell lysates of siRNA-depleted cell were analyzed by western blotting against PTEN and HSP70 as indicated. Error bar = StdDev, N = 3 (* p<0.05, ** p<0.01). [C] NIH/3T3 cells were siRNA depleted for GAPDH, tubulin tyrosine ligase (TTL) or PTEN and treated with nocodazole (0.66 μM, 30 min). Representative images of cells immunostained against detyrosinated tubulin (red), total tubulin (green) and DNA (blue). [D] NIH/3T3 cells were siRNA depleted for GAPDH or PTEN. Representative image of cells immunostained against acetylated tubulin (green), detyrosinated tubulin (red) and DNA (blue). [E] Mean intensity of acetyl or deTyr positive cells. Error bar = StdDev, N = 3 (total of > 150 cells each, * p<0.05, ** p<0.01, *** p<0.001). Scale bars, 10 μm. (TIF) [file pone.0193257.s001.tif]

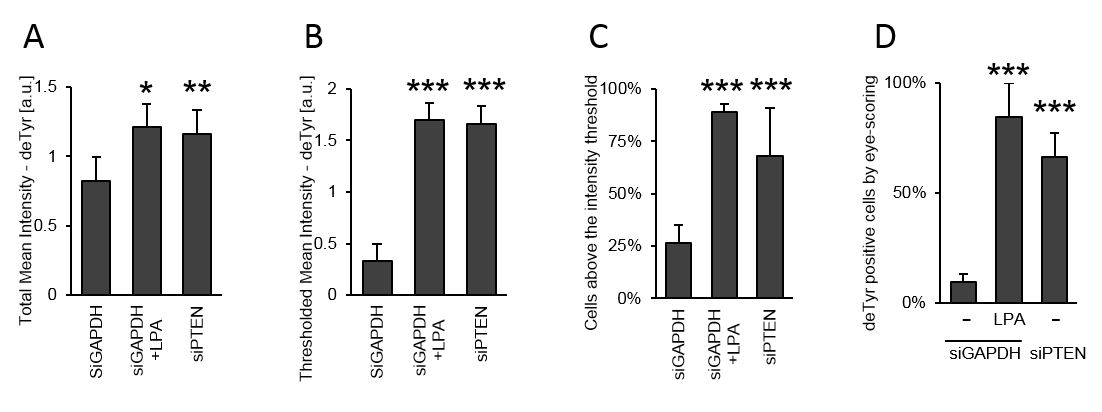

Supplement: S2 Fig — NIH/3T3 cells were siRNA depleted of GAPDH (control) or PTEN, serum-starved and treated with 10 μM LPA (positive control) as indicated. Cells were immunostained for detyrosinated tubulin (deTyr) and the fluorescence signal of each cell was measured. [A] Normalized average intensity of deTyr channel per cell without threshold. [B] Normalized average intensity of deTyr channel per cell with constant threshold. [C] Percentage of cells above the intensity threshold. [D] Percentage of deTyr positive cells by eye-scoring as in Fig 1B. N = 5 (total of >60 cells each). * p<0.05, ** p<0.01, *** p<0.001. (TIF) [file pone.0193257.s002.tif]

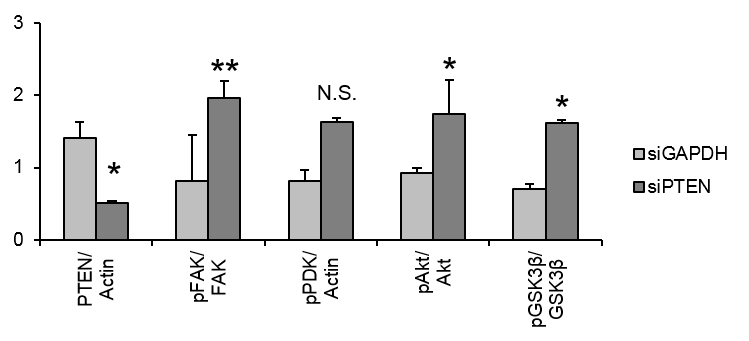

Supplement: S3 Fig — NIH/3T3 cells were siRNA depleted for GAPDH (control) or PTEN and serum depleted overnight. Cell lysates were analyzed by western blotting against PTEN, phospho-FAK (Y397), total FAK, phospho-PDK1, phospho-Akt (Ser473), total Akt, phospho-GSK3β, total GSK3β and β-Actin. Quantification of 3 independent experiments. Error bar = StdDev, N = 3 (* p<0.05, ** p<0.01). (TIF) [file pone.0193257.s003.tif]

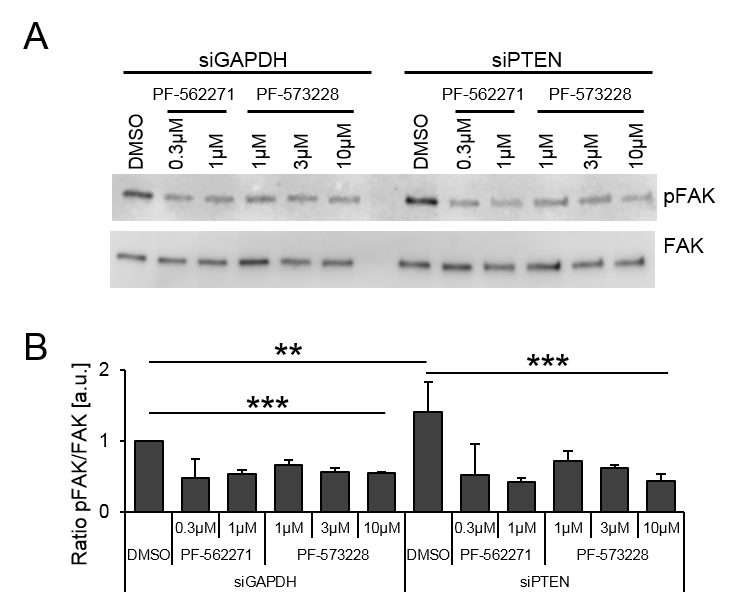

Supplement: S4 Fig — [A, B] NIH/3T3 cells were siRNA depleted against GAPDH or PTEN and treated with FAK inhibitors (PF-562271, PF-573228) as indicated and then seeded on fibronectin-coated coverslips for 2 h. [A] Cell lysates were analyzed by western blotting against pFAK and FAK. [B] Quantification of the western blot signal (ratio pFAK/FAK). Error bar = StdDev, N = 3 (** p<0.01, *** p<0.001). (TIF) [file pone.0193257.s004.tif]

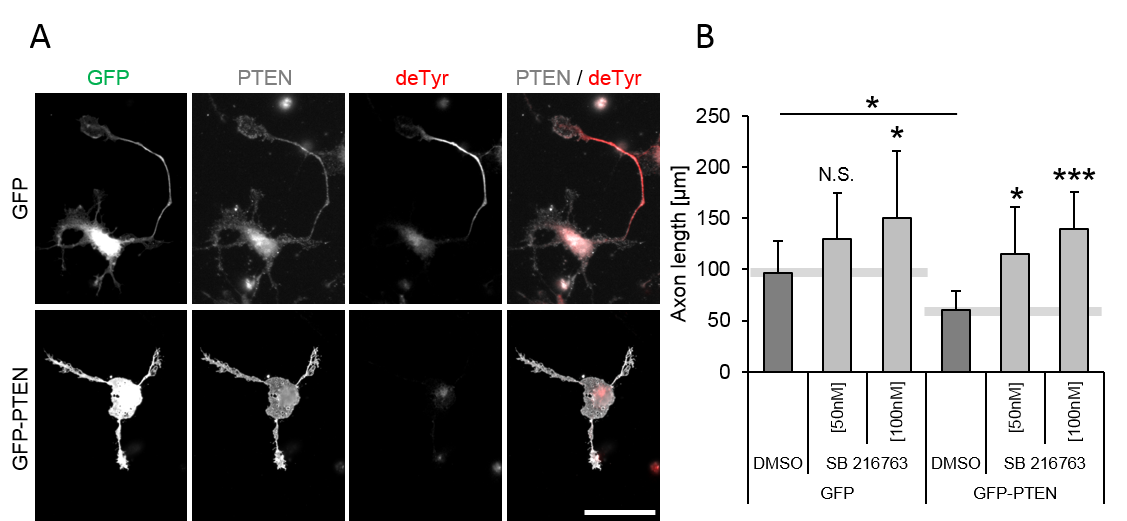

Supplement: S5 Fig — Hippocampal neurons isolated from E16 mice were transfected with GFP or GFP-PTEN at DIV 0, seeded on PLL coated coverslips and fixed at DIV 3. [A] Representative images of neurons immunostained against GFP (green), PTEN (grey) and detyrosinated tubulin (deTyr, red). Scale bar, 50 μm. [B] Quantification of axon length in cells transfected with GFP or GFP-PTEN cells treated with GSK3β inhibitor (SB216763 [50 nM, 100 nM]) as indicated. Error bar = StdDev, N = 4 (total of >40 cells each, * p<0.05, *** p<0.001). (TIF) [file pone.0193257.s005.tif]

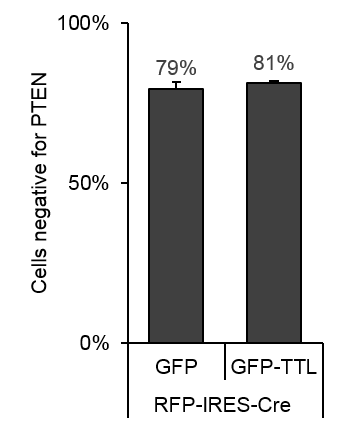

Supplement: S6 Fig — Hippocampal neurons isolated from PTENflox/flox E16 mice were transfected with RFP-IRES-Cre (PTEN-null), GFP or GFP-tubulin tyrosine ligase (TTL) at DIV 0, seeded on PLL coated coverslips and fixed at DIV 3. After fixation, cells were immunostained against GFP and endogenous PTEN. The knockdown efficiency was assayed by scoring PTEN negative cells (low fluorescence intensity in the PTEN channel) in GFP positive cells. Error bar = StdDev, N = 3 (total of >30 cells each). This analysis was used in quantifying detyrosination in Fig 3. (TIF) [file pone.0193257.s006.tif]

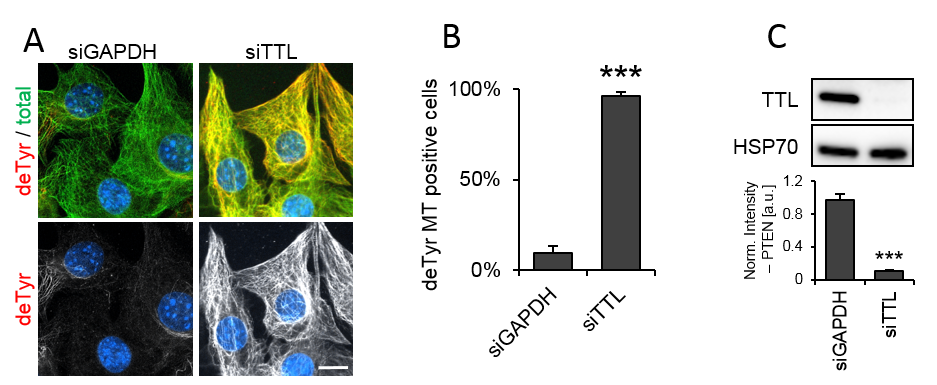

Supplement: S7 Fig — [A] NIH/3T3 cells were siRNA depleted of GAPDH (control) or tubulin tyrosine ligase (TTL) and serum depleted for 1 day. Representative image of cells immunostained against detyrosinated tubulin (red), total tubulin (green) and DNA (blue). Scale bar, 10 μm. [B] Cells were siRNA depleted as indicated. Percentage of detyrosinated microtubules positive cells. [C] Representative western blot of siRNA depleted cells as indicated. Error bar = StdDev, N = 4 (total of >150 cells each, *** p<0.001). (TIF) [file pone.0193257.s007.tif]

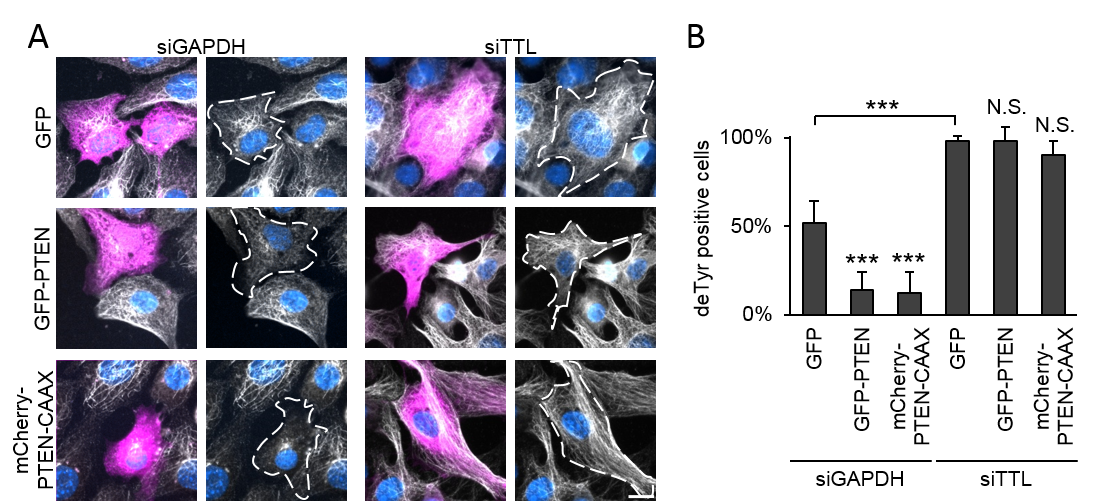

Supplement: S8 Fig — [A] NIH/3T3 cells were siRNA depleted of GAPDH (control) or tubulin tyrosine ligase (TTL) and transfected with GFP, GFP-PTEN or mCherry-PTEN-CAAX and seeded on fibronectin-coated coverslips. Representative images of cells immunostained against detyrosinated tubulin (white), GFP/mCherry (magenta) and DNA (blue). Scale bar, 10 μm. [B] Cells were siRNA depleted as indicated. Percentage of detyrosinated microtubules positive cells. Error bar = StdDev, N = 3 (total of >100 cells each, *** p<0.001). (TIF) [file pone.0193257.s008.tif]
